# Automated determination of 5-hydroxyindolylacetic acid in urine by high-performance liquid chromatography

**DOI:** 10.1155/S1463924682000182

**Published:** 1982

**Authors:** J. P. Garnier, B. Bousquet, C. Dreux

**Affiliations:** Laboratoire de Biochimie Hôpital Saint-Louis 2 place du Dr Fournier Paris 75010 France


Journal of Automatic Chemistry, Volume 4, Number 2 (April–June 1982), pages 65–68

### Automated determination of 5-hydroxyindolylacetic acid in urine by high-performance liquid chromatography

**J. P. Garnier, B. Bousquet and C. Dreux**

*Laboratoire de Biochimie, Hôpital Saint-Louis, 2 place du Dr Fournier, 75010 Paris, France*

5-hydroxyindolylacetic acid (5-HIAA), a major serotonin catabolite, is eliminated in urine. In carcinoid tumours, 95% of which occur in the small intestine, large quantities of serotonin are secreted by the entero-chromaffin cells of Kultchizky-Masson. Early detection of these tumours is essential, since unless they are surgically treated in the initial stages then their prognosis is serious. Early detection can be ensured by measuring blood serotonin and urinary 5-HIAA [1].

Systematic determination of urinary 5-HIAA needs a quick, reliable and automated technique. Neither the α-nitrosonaphtol colorimetric method [2] nor spectrofluorometry after ethyl acetate extraction [3] are suitable: the first lacks specificity and the second is a lengthy and tricky process. Both these methods are manual. Low-pressure chromatography techniques are time-consuming (5-HIAA retention time is over 30 min.) and they are either insufficiently sensitive or unreliable [4, 5, 6]. Reverse-phase separation chromatography also lacks sensitivity and specificity [7], unless it is preceded by manual extraction which, again, is often time-consuming and reduces the technique's precision. High-performance liquid chromatography (HPLC), however, allows fast separation [8].

The method the authors propose rests on the following principle: 5-HIAA is separated from the other metabolites by anion exchange HPLC at pH 2·30, this permits fast, efficient separation. Continuous-flow spectrofluorometric detection is carried out at pH 7·0, at excitation and fluorescence wavelengths of 301 and 338 nm respectively. Chromatography and detection are continuously coupled, in accordance with a process which was worked out in the authors' laboratory [9, 10] and permits optimalization of 5-HIAA pH readings. The use of an automated injector increases precision and allows large numbers of samples to be handled.

### Materials and methods

### *Apparatus*

(1) High-performance liquid chromatograph Chromatem 38 (Touzart Matignon).
(2) Column: Whatman Partisil SAX 10 μm, 250 × 4·7 mm (i.d.) and precolumn 150 × 4·7 mm.
(3) Peristaltic-pump, fluorometer and recorder (Technicon Autoanalyzer II).
(4) Automated sampler and injector (Micromeritics 725 Coultronics).

### *Reagents and standard*

The mobile phase (R1) consists of a buffer solution ($NH_4H_2PO_4$, $5 \times 10^{-3}$ mol/l) adjusted to pH 2·30 with concentrated $H_3PO_4$. R2 is a phosphate buffer solution at pH 7·0, 0·1 mol/l ($Na_2HPO_4$, $2H_2O$ 8·7 g/l and $KH_2PO_4$ 5·6 g/l). R1 and R2 keep for three months at +4°C. R3 is sulphosalicyclic acid solution (500 g/l) in distilled water. The 250 μmol/l 5-HIAA standard is prepared with 0·01 mol/l HCl from a stock solution containing 2·5 mmol/l 5-HIAA in 0·01 mol/l HCl. The standard keeps two weeks and the stock solution keeps three months, both at +4°C in brown flasks. The internal standard is a 2 mmol/l N-acetyltryptophan (NAT) solution in 0·1 mmol/l HCl. It keeps for three months at +4°C in dark bottles.

### *Preparation of urine samples and determinations procedures*

Urine was collected over a 24 h period in 5 ml of hydrochloric acid (10 mol/l). During the day before collection, subjects were asked to avoid some foods, especially bananas and grapefruit, and drugs like reserpine, fluorouracile, heparin, isoniazide, methyldopa, IMAO, corticotrophin and metamphetamine, which are liable to change the *in vivo* urinary concentrations of 5-HIAA.

5 ml urine was acidified by adding 50 μl of sulphosalicyclic acid solution (R3). The preparation was then vortex-stirred for 15 s, centrifuged when necessary and filtered.

Determinations were made as follows: for standardization, each subject's urine was submitted to double overloading with known amounts of 5-HIAA in the presence of an internal standard (NAT). An external standard was used for each series of determinations, for the sole purpose of verifying the fluorescence yield of overloading. When an automatic injector is available, the amounts indicated in table 1 can be introduced directly into the sampling vials. 50 μl of each sample was automatically injected every 7 min.

A diagram of the apparatus used is shown in figure 1; the flow rate was 1·50 ml/min. An example of a urinary chromatogram is presented in figure 2.

### *Calculation of urine concentrations*

The heights of the peaks corresponding to 5-HIAA were measured (A for U [urine]; B for U + 25 μmol/l; C for U + 50 μmol/l). The peaks corresponding to NAT were also measured (a for U; b for U + 25 μmol/l; and c for U + 50 μmol/l).

*S* is the corrected height for an average overload of 5-HIAA equivalent to 25 μmol/l and was calculated as follows:

$$S = \frac{1}{2}\left[\frac{1}{2}\left(\frac{C}{c} - \frac{A}{a}\right) + \left(\frac{B}{b} - \frac{A}{a}\right)\right]$$

0142 0453/0402 0065 $05·00 © 1982 Taylor & Francis Ltd

The final urine concentration was:

$$C \ \mu mol/l = \frac{25}{S} \times \frac{A}{a} \mu mol/l,$$

$$C \ \mu mol/24\,h = C\,\mu mol/l \times \text{urinary flow } 1/24\,h,$$

$$C \ \mu mol/mmol \text{ of creatinine} = \frac{C\,\mu mol/l}{C \text{ creatinine mmol/l}}.$$

### Results and discussion

### *Choice of experimental conditions*

pH and mobile phase molarity were studied within limits compatible with the stationary phase. The pH was varied from 1·50 to 7·00, and the molarity from $10^{-4}$ mol/l to 1 mol/l.

Optimal conditions were at pH 2·30 and a molarity of $5 \times 10^{-3}$ mol/l. Under these conditions 5-HIAA was well separated from its metabolites (tryptophan or Trp, hydroxy-5-tryptamine or 5-HT and hydroxy-5-tryptophan or 5-HTP) (see figure 2).

*Table 1. Sampling conditions for the automatic injector.*

| | External standard | U | U+ 25 μmol/l | U+ 50 μmol/l |
|---|---|---|---|---|
| NAT (2 mmol/l) (internal standard) | 100 μl | 100 μl | 100 μl | 100 μl |
| 5-HIAA (250 μmol/l) | 50 μl | — | 50 μl | 100 μl |
| Mobile phase | 550 μl | 100 μl | 50 μl | 500 μl |
| Acidified urine | — | 500 μl | 500 μl | 500 μl |
| Vortex-stir for 15 s | | | | |

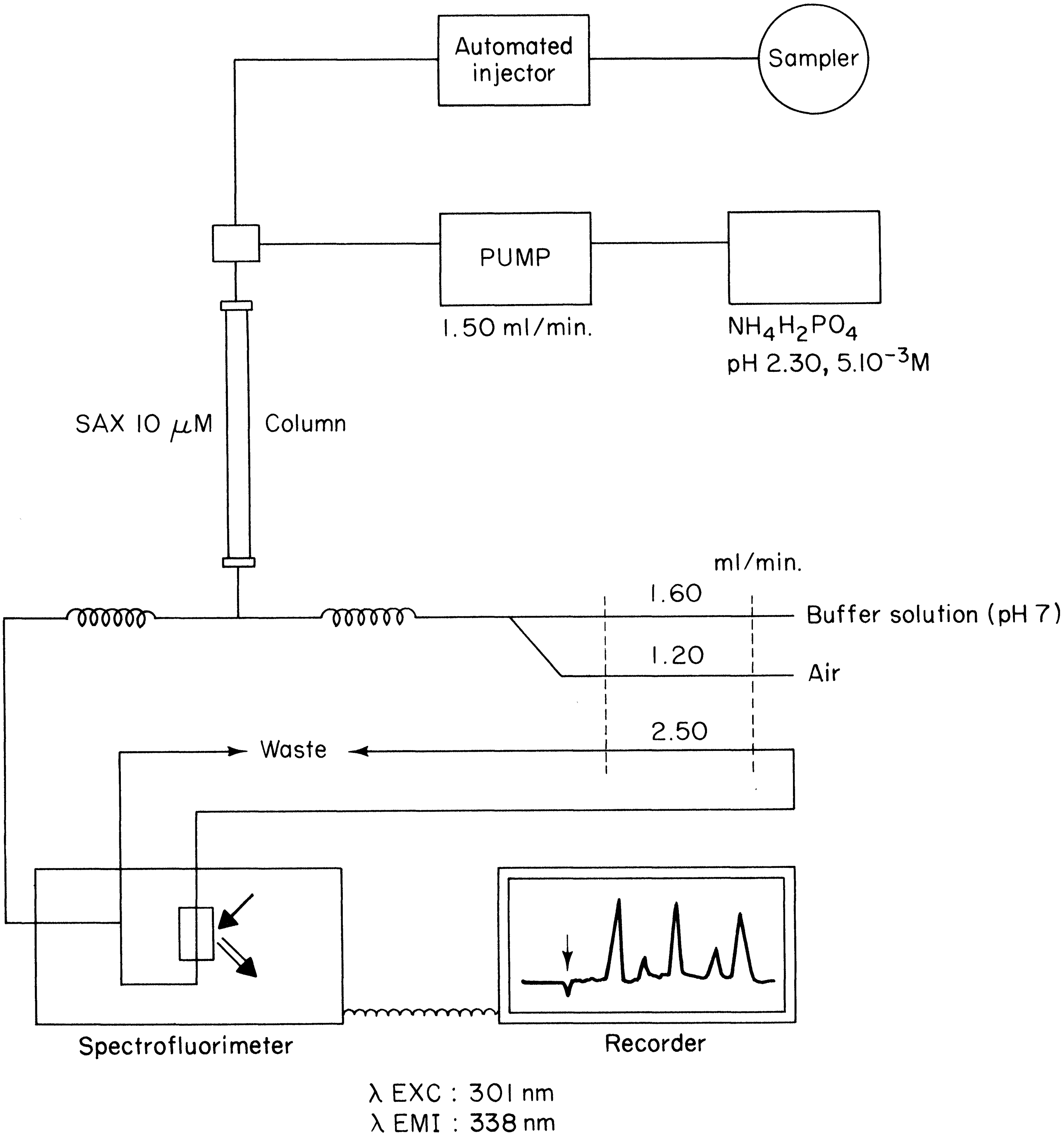

*Figure 1. Chromatographic and analytical system for automated determination of 5-HIAA.*

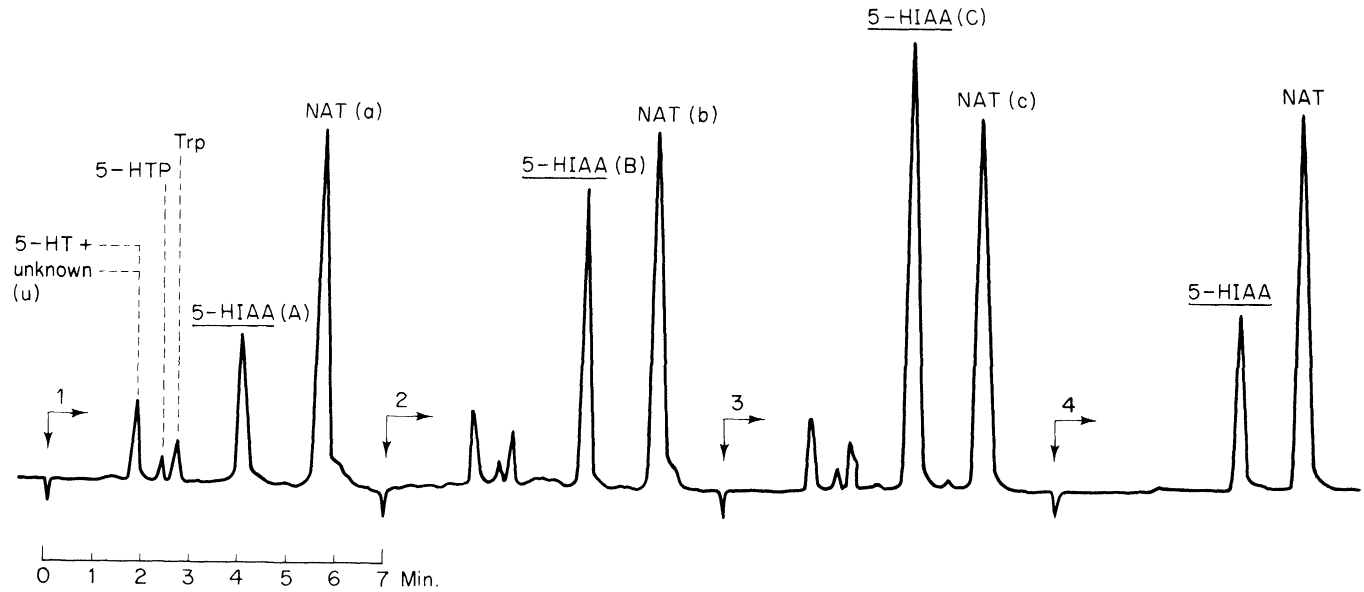

*Figure 2. Chromatograms of urine (1), urine + 25 μmol/l 5-HIAA (2), urine + 50 μmol/l 5-HIAA (3), and 25 μmol/l 5-HIAA standard (4).*

*A, B and C = height of peaks corresponding to 5-HIAA; a, b, c = height of peaks corresponding to NAT; 5-HT = 5-hydroxytryptamine; 5-HTP = 5-hydroxytryptophan; Trp = tryptophan.*

The fluorometric spectrum for 5-HIAA was verified with our apparatus and maximal excitation and emission wavelengths were found to be 301 and 338 nm respectively. Fluorometric pH readings for 5-HIAA were studied between 2 and 10, and the optimal pH was 7·0 (at this pH the fluorescence intensity of 5-HIAA increased fourfold compared to that at pH 2·30).

Chromatography requires the use of an internal standard to eliminate uncertainty regarding the volumes injected. The spectrofluorometric characteristics of NAT enable satisfactory detection under the conditions described above. Its retention time is 1 min. 30 s longer than that of 5-HIAA.

Fluorometric detection requires standardization by overloading. The overloading yield ($R$) was established for each determination. It enabled us to determine the presence of either a fluorescence activator ($R > 100\%$) or inhibitor ($R < 100\%$). In 99% of cases the overload yield was between 90 and 105%. Minor interferences were then automatically corrected by calculating the overload.

### Analytical results

The authors found a detection limit of 1 pmol which agreed with the results in the literature [6, 7, 8].

Linearity was satisfactory up to 250 μmol/l, i.e. about 10 times the usual mean value.

As regards within-run precision, 36 successive measurements of the same sample (28·5 μmol/l) showed the following coefficients of variation (CV): 2·90% for manual injection, 1·30% for automatic injection with no internal standard, and 0·72% for automatic injection with an internal standard.

Sample preparation is simple and quick (a few minutes) and one sample can be injected every 7 min. Since injection is automatic, the apparatus requires no supervision.

The absence of contamination was verified by measuring alternately low standards (5 μmol/l) and high standards (100 μmol/l). There was no significant difference ($p < 0{\cdot}05$) between the low standard values, whether they preceded or followed the high values.

The specificity of 5-HIAA determinations was ensured by combining chromatographic separation and spectrofluorometric detection, as follows:

(1) Chromatography permitted separation of 5-HIAA from its metabolic precursors present in urine.
(2) Spectrofluorometry was carried out at maximum wavelengths and at the optimum pH for 5-HIAA.

Absence of interference was tested on certain drugs, particularly those interfering in the α-nitrosonaphtol technique. For each test, the drug was added to urine at a concentration corresponding to the maximum therapeutic dose: 6 g/l acetylsalicyclic acid, 150 mg/l of promethazine, 200 mg/l chlortetracycline, 2·5 g/l sulfamethoxazole, 150 mg/l chlorpromazine and 100 mg/l prochlorperazine. At these concentrations, unlike the colorimetric method, no significant interference was shown.

Comparison of the direct method suggested was tested with (*a*) the same method but preceded by ethyl acetate extraction; (*b*) with a spectrofluorometric reference method.

### (*a*) *Ethyl acetate extraction*

This technique is long but has great specificity, since 5-HIAA is the only metabolite extracted from urine in large amounts, see figures 3 (*a*) and (*b*). The extraction yield was $94 \pm 5\%$ ($N = 54$). A schematic indication of the procedure adopted is given in table 2. 50 μl samples were injected into the chromatograph under the conditions specified above. Comparison with the direct method (without extraction) gave the following results: correlation line: $y = 0{\cdot}97\ x + 0{\cdot}09$; correlation coefficient: $r = 0{\cdot}992$ ($N = 54$). These results indicate that chromatographic separation specificity is sufficient in most cases and does not require prior extraction.

### (*b*) *Spectrofluorometric reference method*

68 determinations of urinary 5-HIAA were compared using a manual spectrofluorometric technique. The technique was preceded by extraction [3]. Statistical study showed a good correlation: correlation line: $y = 0{\cdot}89\ x + 1{\cdot}88$; correlation coefficient: $r = 0{\cdot}96$; $t$ (Student) = 38·9 > theoretical $t = 3{\cdot}44$ ($p < 0{\cdot}001$). Reference values, determined in 112 healthy subjects, were $25 \pm 20$ μmol/24 h (m $\pm 2\sigma$, $p < 0{\cdot}05$). These values agree with those found by several authors, see references [1–5] and [11–14].

Urinary 5-HIAA was also determined in 19 cases with carcinoid tumours. Values ranged from 94 to 4350 μmol/24 h. A

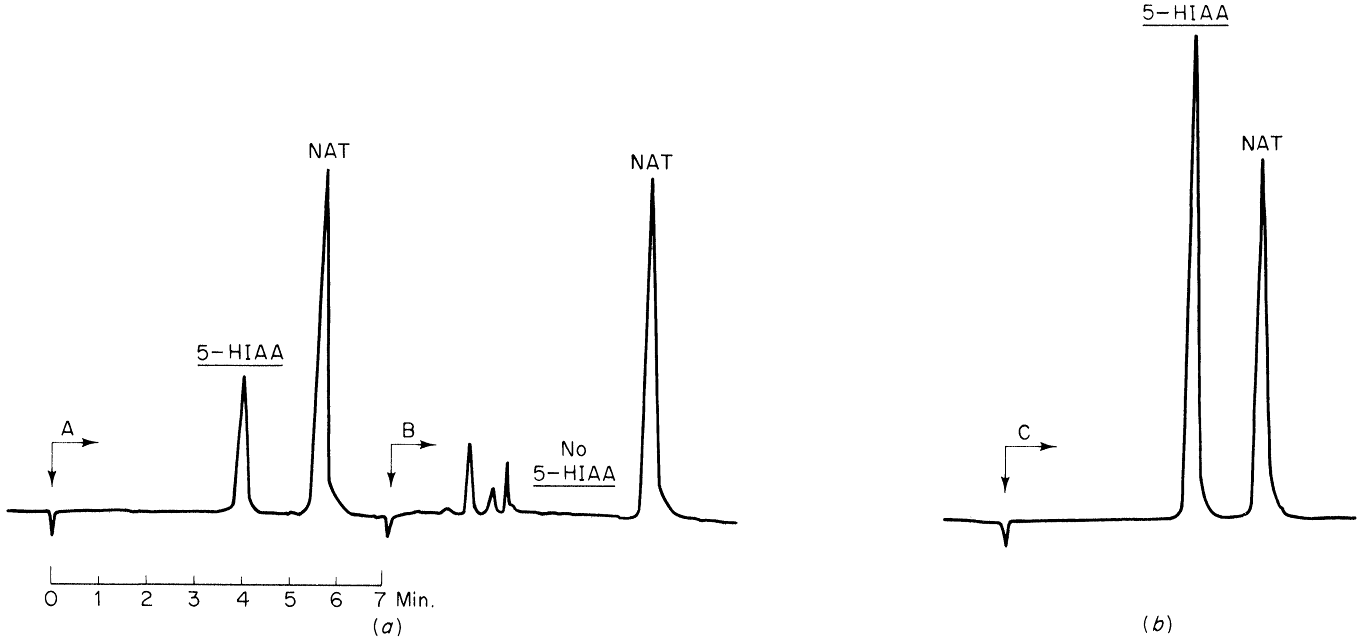

*Figure 3 (a and b). Chromatograms of ethyl acetate extract (A), aqueous phase extract (B), and 1 μl of carcinoid urine (C).*

*Table 2. Procedure adopted for the ethylacetate extraction method.*

| | External standard 5-HIAA 25 μmol/l | U | U+ 25 μmol/l | U+ 50 μmol/l |
|---|---|---|---|---|
| | In 5 ml glass tubes | | | |
| NaCl | 100 mg | 100 mg | 100 mg | 100 mg |
| HCl 6 N | 20 μl | 20 μl | 20 μl | 20 μl |
| Acidified urine | 0 | 500 μl | 500 μl | 500 μl |
| HCl 0·01 N | 550 μl | 100 μl | 50 μl | — |
| 5-HIAA 250 μmol/l | 50 μl | — | 50 μl | 100 μl |
| Ethyl acetate | 1000 μl | 1000 μl | 1000 μl | 1000 μl |
| Stopper the tubes, vortex-stir for 3 min.; centrifuge when necessary, remove the organic phase and treat as follows: | | | | |
| Organic phase | 500 μl | 500 μl | 500 μl | 500 μl |
| Evaporate to dryness in waterbath at 31°C with a vacuum pump and resuspend the residue | | | | |
| Mobile phase | 500 μl | 500 μl | 500 μl | 500 μl |
| NAT 2 mmol/l | 100 μl | 100 μl | 100 μl | 100 μl |

chromatogram of carcinoid tumour urine is shown in figure 2. Results were excellently correlated with the reference technique [3] and agreed with clinical and anatomopathological observations.

### Conclusion

High performance liquid chromatography, combined with continuous-flow fluorometric detection at an optimized pH, permits quick specific measurements of urinary 5-HIAA. Since the method is both completely automated and simple, it can be used for systematic determinations in carcinoid tumour detection. Its specificity enables ethyl acetate extraction to be avoided and its precision and sensitivity make it a particularly reliable method for use in clinical biochemical laboratories.

### References

1. Dreux, C., Bousquet, B. and Halter, D., *Annales de Biologie Clinique*, **31** (1973), 283.
2. Dreux, C. and Delauneux, B., *Presse Médicale*, **49** (1964), 2925.
3. Bousquet, B. and Launay, J. M., *Pharmacien Biologiste*, No. 80 (1972).
4. Yoshida, A., Yamazaki, T. and Sakai, T., *Clinica Chimica Acta*, **77** (1977), 95.
5. Brown, H. H., Rhindress, M. C. and Griswold, R. E., *Clinical Chemistry*, **17** (1971), 92.
6. Chilcote, D. D., *Clinical Chemistry*, **18** (1972), 1376.
7. Graffeo, A. P. and Karger, B. L., *Clinical Chemistry*, **22** (1976), 184.
8. Beck, O., Palmskog, G. and Hultman, E., *Clinica Chimica Acta*, **79** (1977), 149.
9. Garnier, J. P., Bousquet, B. and Dreux, C., *Feuillets de Biologie*, **106** (1979), 135.
10. Garnier, J. P., Bousquet, B. and Dreux, C., *Analusis*, **7** (1979), 355.
11. Pierce, C., *American Journal of Clinical Pathology*, **30** (1958), 230.
12. Arterberry, J. D. and Conney, M. P., *Clinical Chimica Acta*, **17** (1967), 431.
13. Korf, J. and Valkenburg-Sikkema, T. S., *Clinica Chimica Acta*, **26** (1969), 301.
14. Yamaguchi, Y. and Hayashi, C., *Clinical Chemistry*, **24** (1978), 149.

